# Baseline FDG-PET Brain hypometabolism as a predictive biomarker of cognitive decline and Alzheimer’s disease risk

**DOI:** 10.1016/j.jnha.2026.100823

**Published:** 2026-03-11

**Authors:** Ayman S. Alhasan, Mustafa S. Alhasan, James Milburn, Hadeel A. Ghunaim, Mohammad Khalil, Abdullah Almaghraby, Omar Alharthi, Seham Hamoud, Muhammed Amir Essibayi, Yasir Hassan Elhassan, Fabricio Feltrin, Sumit Singh, Dhairya A Lakhani, Ahmed Y. Azzam

**Affiliations:** a Department of Internal Medicine, College of Medicine, Taibah University, Madinah, Saudi Arabia; b Consultant Teleradiologist, Teleradiology Solutions, 22 Llanfair Rd UNIT 6, Ardmore, PA 19003, United States of America; c The University of Queensland Medical School, Ochsner Clinical School, New Orleans, Louisiana, United States of America; d Department of Radiology, Ochsner Clinic Foundation, New Orleans, Louisiana, United States of America; e Faculty of Medicine, King Abdulaziz University, Jeddah, Saudi Arabia; f Department of Pediatrics, Umm Al-Qura University, Makkah, Saudi Arabia; g Department of Pediatrics, Taibah University, Madinah, Saudi Arabia; h Montefiore-Einstein Cerebrovascular Research Lab, Montefiore Medical Center, Albert Einstein College of Medicine, Bronx, NY, United States of America; i Department of Neurological Surgery, Montefiore Medical Center, Albert Einstein College of Medicine, Bronx, NY, United States of America; j Department of Basic Medical Sciences, College of Medicine, Taibah University, Madinah, Saudi Arabia; k Division of Radiology – Neuroradiology, University of Texas Southwestern Medical Center, 5323 Harry Hines Blvd, Dallas, TX 75390, United States of America; l Department of Neuroradiology, Rockefeller Neuroscience Institute, West Virginia University, Morgantown, West Virginia, United States of America; m Division of Global Health and Public Health, School of Nursing, Midwifery and Public Health, University of Suffolk, Ipswich, Suffolk, United Kingdom

**Keywords:** Alzheimer's disease, Glucose metabolism, Brain glucose hypometabolism, Cognitive decline, Positron emission tomography

## Abstract

**Introduction:**

Brain glucose hypometabolism precedes cognitive decline in Alzheimer's disease, however its role in determining long-term cognitive trajectories remains under studied in current literature in a clear manner. We investigated whether baseline brain glucose metabolism predicts cognitive decline rates and disease conversion risk in a large longitudinal cohort.

**Methods:**

We analyzed 4,732 participants (1,685 with longitudinal cognitive data) from the Alzheimer's Disease Neuroimaging Initiative (ADNI) with fluorodeoxyglucose positron emission tomography (FDG-PET), Mini-Mental State Examination (MMSE), and Alzheimer's Disease Assessment Scale (ADAS) measurements over ten years. Mixed-effects models investigated time × brain glucose metabolism interactions on cognitive decline. Cross-validation assessed predictive accuracy for mild cognitive impairment and Alzheimer's disease conversion.

**Results:**

Brain glucose metabolism was found to modulate the cognitive decline rates (time × FDG interaction: MMSE β = 0.746, P-value <0.001; ADAS β = −1.595, P-value <0.001). High versus low glucose metabolism demonstrated 1.49 MMSE points/year and 3.19 ADAS points/year protection. Among cognitively normal participants, low glucose metabolism increased Alzheimer's disease conversion risk four-fold (incidence rate ratio = 3.79, 95%CI: 2.94–4.88). Predictive models achieved high accuracy for Alzheimer's disease conversion (AUC = 0.826) with good calibration (Brier score = 0.092). High-metabolism individuals showed essentially stable cognition over ten years follow-up duration as evident in the ADNI dataset.

**Conclusions:**

We found that brain glucose metabolism is a notable determinant of cognitive decline progression, especially for Alzheimer’s disease risk, providing quantifiable metabolic protection against decline. Our results demonstrate brain glucose hypometabolic findings from baseline FDG-PET as a precision biomarker for therapeutic stratification and support further interventions for cognitive preservation for further research, validation and development purposes that should be further evaluated and investigated in further clinical trials that may lead to better preventive and therapeutic benefits for cognitive functions preservation and prevention of cognitive decline process in high-risk individuals.

## 1 Introduction

Brain glucose metabolism represents an energy currency supporting cognitive function, with newer literature evidence that metabolic dysfunction contributes and drives neurodegeneration in cognitive decline and Alzheimer's disease. Fluorodeoxyglucose positron emission tomography (FDG-PET) is a unique imaging modality that can capture patterns and early changes of glucose hypometabolism and brain metabolic activity several years before symptom onset, suggesting that brain energy failure constitutes a core pathophysiological mechanism rather than a downstream consequence of neurodegeneration. However, the extent to which individual differences in metabolic brain changes and dysfunction determine long-term cognitive trajectories and outcomes remains limited in current literature evidence as it is still considered a new hypothesis of neurodegeneration pathophysiology and further warrants detailed evaluation and investigational studies [[1], [2], [3], [4]].

Current approaches to dementia prediction rely on cognitive assessments and structural neuroimaging, which capture disease processes relatively late in the pathological phases. In contrast, the recent literature evidence demonstrates a new promising role for early detection of brain metabolic activity changes that could impact the immediate functional capacity of neural networks to maintain cognitive operations under metabolic stress. This hypothetical biological process may therefore provide superior prognostic information for identifying individuals at risk for cognitive decline and those likely to maintain cognitive resilience that could lead to new innovation and progression in the next years for cognitive disorders and neurodegeneration [[5], [6], [7], [8], [9]].

The Alzheimer's Disease Neuroimaging Initiative (ADNI) is large longitudinal, multicenter, observational database collecting big data from multiple institutions according the United States of America for neuroimaging data with follow-up from different neuroimaging with biological biomarkers for research and development purposes, especially for Alzheimer’s disease that offers us a valuable opportunity to evaluate and investigate the longitudinal relationship between brain structural imaging modalities, brain metabolic activity from imaging and biomarker data, as well as clinical data with cognitive outcomes across the disease spectrum [[10], [11], [12], [13]].

Understanding the quantitative relationship between brain glucose metabolism and cognitive decline outcomes has multiple implications for precision medicine to dementia prevention. If brain glucose metabolism indeed determines cognitive fate, then metabolic biomarkers could allow early identification of at-risk individuals, guide therapeutic stratification, and serve as targets for precise and personalized targeted interventions for high-risk individuals. In addition to that, further validation and demonstration of predictive accuracy would facilitate clinical trial development strategies and help us to further test and validate biomarker-guided decisions [[14], [15], [16], [17], [18]].

We hypothesized that baseline brain glucose metabolism would predict the rate of cognitive decline over time, with preserved metabolism conferring quantifiable protection against cognitive deterioration. Also, we anticipated that FDG-based models would demonstrate superior predictive accuracy for disease risk conversion events, that could be achieving levels suitable for further testing and implementation purposes for cognitive decline and Alzheimer’s disease.

## 2 Methods

### 2.1 Study design and participants

This longitudinal observational study analyzed data from ADNI. ADNI is a longitudinal multicenter observational study launched in 2004, designed to develop and validate clinical, imaging, genetic, and biochemical biomarkers for the early detection and tracking of Alzheimer's disease [[19], [20], [21], [22], [23], [24], [25]].

ADNI enrolls participants aged 55–90 years across around 60 sites in the United States and Canada, including cognitively normal individuals, those with mild cognitive impairment, and patients with early Alzheimer's disease dementia. Key inclusion criteria include Mini-Mental State Examination scores between 24–30 for cognitively normal participants and 20–26 for MCI, with specific memory performance thresholds on the Logical Memory II subscale. Exclusion criteria include neurological disease other than Alzheimer's disease, major psychiatric disorders, substance abuse, and contraindications to magnetic resonance imaging (MRI). It is important to note that ADNI participants are predominantly non-Hispanic White (around 90%), highly educated (mean around 16 years), and recruited primarily from academic medical centers, which may limit generalizability to more different populations.

**Fig. 1:**
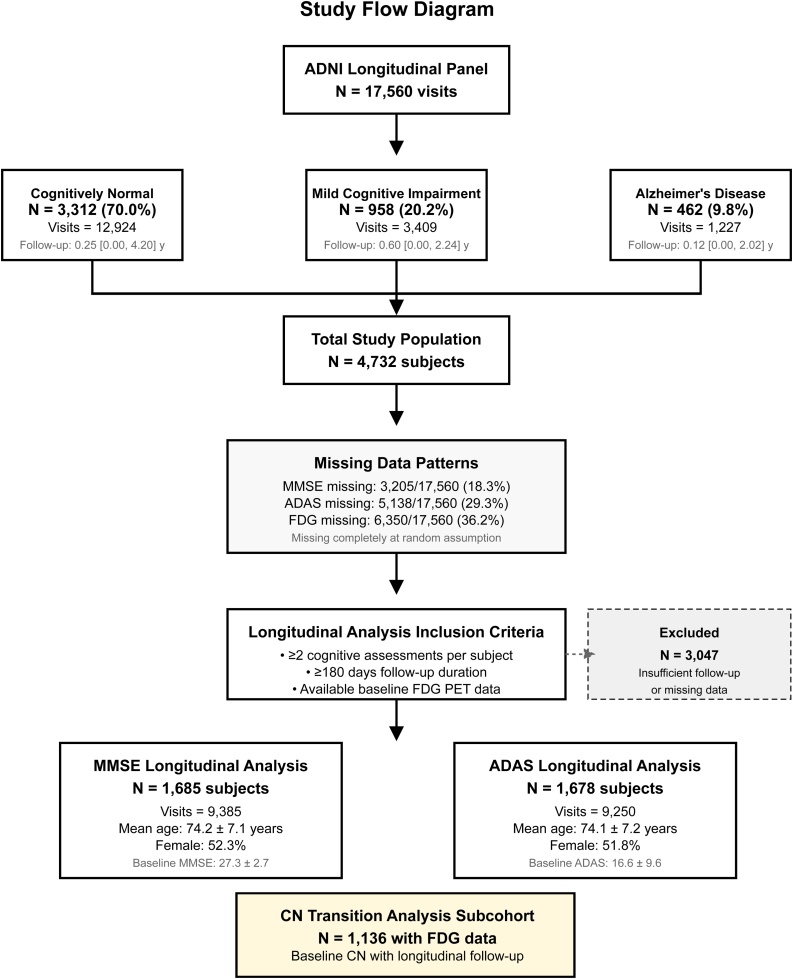
Study pipeline flowchart diagram.

In our study, we included all participants with baseline FDG-PET neuroimaging and at least two cognitive assessments spanning a minimum of 180 days follow-up through August 2025. We followed the Strengthening the Reporting of Observational Studies in Epidemiology (STROBE) guidelines for reporting longitudinal cohort studies [26,27] as demonstrated in Fig. 1.

Participants were classified at baseline into cognitively normal (CN), mild cognitive impairment (MCI), or Alzheimer's disease groups using the sorted ADNI diagnostic criteria. All participants provided written informed consent, and the study was approved by institutional review boards at participating sites in which ADNI collected their data and findings from. Data access was obtained through the ADNI database following formal application and approval process with compliance to all ethical and regulatory process requirements.

### 2.2 Brain glucose metabolism assessment

FDG-PET imaging was performed according to the ADNI protocols using harmonized acquisition parameters across participating sites. Images were preprocessed using the University of California, Berkeley processing pipeline with 8 mm smoothing kernels applied for optimal signal-to-noise characteristics. Brain glucose metabolism was quantified using the MetaROI composite score, which represents a standardized region-of-interest approach combining metabolically sensitive brain regions into a single summary measure.

For participants with multiple FDG-PET scans, baseline values were defined as the earliest available measurement, preferentially selecting screening or baseline visit data when available. Raw MetaROI values were converted to standardized z-scores relative to the study population to facilitate interpretation and proper feasible translation. Our utilized approach was utilized and implemented to allow direct comparison of metabolic effects across different analytical methods and settings and provides meaningful effect size estimates.

### 2.3 Cognitive outcome measures

Cognitive function was assessed using two well-validated instruments administered according to ADNI protocols. The Mini-Mental State Examination (MMSE), developed by Folstein et al. in 1975 [28], is a 30-point cognitive screening tool requiring approximately 5−10 min to administer. It assesses orientation, registration, attention, recall, and language, with scores ranging from 0 to 30 where higher scores indicate better function. Clinically, scores of 24–30 suggest normal cognition, 18–23 indicate mild impairment, and below 17 indicate severe impairment. For dementia detection, scores ≤24 result in around 87% sensitivity and 82% specificity [29]. Limitations include ceiling effects in highly educated individuals, limited sensitivity to mild cognitive impairment, and performance influenced by age and educational attainment.

The Alzheimer's Disease Assessment Scale-Cognitive Subscale (ADAS-Cog), developed by Rosen et al. in 1984 [30], is a more comprehensive assessment evaluating memory, language, and praxis domains. The ADAS-Cog consists of 11 items with scores ranging from 0 to 70 points, where higher scores indicate greater impairment (opposite direction to MMSE). Administration requires 30−60 min and trained raters. The ADAS-Cog is considered the gold standard cognitive outcome in Alzheimer's disease clinical trials and demonstrates greater sensitivity to longitudinal change compared to MMSE [31]. Limitations include time-intensive administration and reduced sensitivity in very early disease stages.

Longitudinal cognitive data were merged with visit dates to calculate precise time intervals from baseline assessments. We constructed a structured and detailed longitudinal panel dataset linking cognitive measurements with FDG-PET data and baseline demographic characteristics. Only participants with at least two cognitive assessments separated by a minimum of 180 days were included to ensure appropriate and accurate longitudinal follow-up for trajectory estimation.

### 2.4 Disease risk conversion outcomes

Disease risk conversion events were defined using longitudinal diagnostic classifications from ADNI patients’ data assessments. For cognitively normal participants at baseline, we identified conversion to MCI or Alzheimer’s disease using time-to-event estimations. Conversion pathways were categorized as: remaining cognitively normal, progressing to MCI only, progressing from MCI to Alzheimer’s disease, or converting directly from normal cognition to Alzheimer’s disease. These pathway definitions were intended to help us to capture and identify characterization of disease progression patterns and risk stratification based on brain glucose metabolism levels.

Event times were calculated as the interval between baseline assessment and first documented diagnostic change. Participants without conversion events were censored at their last available assessment. This approach was implemented to ensure proper robust time-to-event data suitable for survival analysis and incidence rate calculations while accounting for differential follow-up durations across participants.

### 2.5 Statistical analysis

In our primary analyses’ endpoints, we utilized linear mixed-effects models to investigate the longitudinal cognitive trajectories as functions of time, baseline brain glucose metabolism, and their interaction. These models included random intercepts and slopes for individual participants to account for within-subject correlation and individual heterogeneity in cognitive trajectories. Fixed effects included time since baseline (continuous), FDG MetaROI z-score (continuous), and their interaction term, which quantifies how brain glucose metabolism modulates the rate of cognitive change over time.

Secondary analyses stratified participants by FDG tertiles (low, middle, high glucose metabolism) to provide interpretable risk categories and facilitate translation to further practice settings. We calculated predicted cognitive slopes for representative FDG levels (−1, 0, +1 standard deviations) to quantify metabolic protection effects in meaningful units.

Model selection utilized information criteria comparisons and residual diagnostics to optimize functional form specifications. We conducted sensitivity analyses using alternative inclusion criteria (requiring ≥3 visits instead of ≥2, or ≥90 days instead of ≥180 days follow-up), different missing data approaches (complete case analysis versus multiple imputation), and multiple estimation methods (mixed-effects models versus ordinary least squares with clustered standard errors) to evaluate result stability. We also assessed nonlinearity by comparing linear time trends to restricted cubic spline models. For cross-validation, we utilized five-fold subject-grouped resampling to prevent optimistic bias while maintaining independence assumptions.

### 2.6 Predictive modeling and validation

For disease risk conversion prediction, we developed logistic regression models using baseline FDG-PET data to predict MCI and Alzheimer’s disease conversion at 12-months, 24-months, and 36-month horizons. Model performance was evaluated using area under the receiver operating characteristic curve (AUC), Brier scores for calibration assessment, and bootstrap confidence intervals (CI) for uncertainty quantification.

Multiple validation methods included subject-grouped cross-validation to prevent data leakage, learning curve to assess overfitting, and temporal validation using different time periods. We calculated sensitivity, specificity, positive predictive value, and negative predictive value at optimal thresholds determined by Youden's index maximization. Statistical significance was assessed using DeLong tests for AUC comparisons and Hosmer-Lemeshow tests for calibration evaluation.

All analyses were conducted using mixed-effects modeling using RStudio software with R version 4.4.2, and Python 3.13 with utilization with the appropriate statistical and computational modeling packages. Statistical significance was defined as P-value less than 0.05 for primary hypotheses, with Bonferroni correction applied for multiple comparisons where appropriate. Effect sizes for cognitive outcomes were interpreted using established minimal clinically important difference (MCID) benchmarks: for MMSE, 1–2 points represents clinically meaningful change in mild cognitive impairment and 2–3 points in mild Alzheimer's disease; for ADAS-Cog, 2–3 points is meaningful in MCI and 3+ points in mild Alzheimer's disease. For discrimination metrics [32,33]., AUC values were interpreted as: 0.5−0.7 poor, 0.7−0.8 acceptable, 0.8−0.9 excellent, and >0.9 outstanding discrimination [34,35].

## 3 Results

### 3.1 Study population and baseline characteristics

Our study included 4,732 participants from the ADNI cohort, with 3,312 cognitively normal (70.0%), 958 MCI (20.2%), and 462 Alzheimer's disease (9.8%) individuals at baseline (Supplementary Table S1). The longitudinal dataset included 1,685 participants with MMSE assessments (9,385 visits; mean 5.6 assessments per participant, range 2–15) and 1,678 participants with ADAS assessments (9,250 visits; mean 5.5 assessments per participant, range 2–15), representing participants with at least two cognitive assessments spanning ≥180 days follow-up. Baseline demographic and clinical characteristics of these analytical samples, stratified by cognitive status, are presented in Table 1.

**Table 1:** Baseline Demographics, Clinical Characteristics, Data Quality, and Longitudinal Brain Glucose Metabolism Effects with Quantified Metabolic Protection.

Characteristic	Overall Study	CN	MCI	AD	MMSE Assessment	ADAS Assessment
**Demographic Characteristics:**						
Age, years, mean ± SD	73.8 ± 7.2	73.2 ± 6.9	74.1 ± 7.4	75.8 ± 7.8	–	–
Female, N (%)	2,344 (49.5%)	1,732 (52.3%)	401 (41.9%)	211 (45.7%)	–	–
Education, years, mean ± SD	16.1 ± 2.7	16.3 ± 2.6	15.9 ± 2.8	15.4 ± 2.9	–	–
Race/Ethnicity	–	–	–	–	–	–
Non-Hispanic White, N (%)	4,212 (89.0%)	2,945 (88.9%)	858 (89.6%)	409 (88.5%)	–	–
Black/African American, N (%)	237 (5.0%)	171 (5.2%)	43 (4.5%)	23 (5.0%)	–	–
Hispanic/Latino, N (%)	142 (3.0%)	98 (3.0%)	29 (3.0%)	15 (3.2%)	–	–
Asian, N (%)	94 (2.0%)	65 (2.0%)	19 (2.0%)	10 (2.2%)	–	–
Other, N (%)	47 (1.0%)	33 (1.0%)	9 (0.9%)	5 (1.1%)	–	–
APOE ε4 carrier, N (%)	1,988 (42.0%)	1,226 (37.0%)	478 (49.9%)	284 (61.5%)	–	–
BMI, kg/m^2^, mean ± SD	27.1 ± 4.8	27.3 ± 4.7	26.8 ± 4.9	26.2 ± 5.1	–	–
**Study Population Flow:**						
Initial panel visits, N	17,560	–	–	–	–	–
Participants meeting eligibility, N	–	–	–	–	1,685	1,678
Final analysis visits, N	–	–	–	–	9,385	9,250
Participants excluded, N	3,047	–	–	–	–	–
**Study Population:**						
Participants, N	4,732	3,312 (70.0%)	958 (20.2%)	462 (9.8%)	1,685	1,678
Total visits, N	17,560	12,924	3,409	1,227	9,385	9,250
Visits per participant, mean ± SD	–	3.90 ± 3.58	3.56 ± 2.80	2.66 ± 1.77	–	–
Follow–up, years, median (IQR)	–	0.25 (0.00, 4.20)	0.60 (0.00, 2.24)	0.12 (0.00, 2.02)	–	–
**Missing Data Patterns:**						
MMSE missing, N (%)	3,205 (18.3%)	–	–	–	–	–
ADAS missing, N (%)	5,138 (29.3%)	–	–	–	–	–
FDG missing, N (%)	6,350 (36.2%)	–	–	–	–	–
**Baseline Cognitive and Metabolic Measures:**						
MMSE score, mean ± SD	–	28.87 ± 1.04	25.21 ± 0.88	20.26 ± 3.40	27.26 ± 2.67	–
ADAS score, mean ± SD	–	11.45 ± 6.05	21.72 ± 7.60	32.22 ± 8.54	–	16.60 ± 9.58
FDG MetaROI z–score, mean ± SD	–	0.31 ± 0.75	–0.41 ± 1.01	–1.18 ± 1.23	0.000 ± 1.000	0.000 ± 1.000
**Data Quality and Reliability:**						
Intraclass correlation coefficient	–	–	–	–	0.593	0.748
Cross-validation RMSE	–	–	–	–	1.43	3.00
Cross-validation MAE	–	–	–	–	1.00	2.19
95% prediction interval coverage, %	–	–	–	–	95.0	94.7
**Mixed-Effects Model Results:**						
Time (years)	–	–	–	–	–0.854 (0.029) [–0.911, –0.798], p<0.001	1.994 (0.061) [1.874, 2.114], p<0.001
FDG MetaROI (z–score)	–	–	–	–	1.364 (0.059) [1.248, 1.480], p<0.001	–5.311 (0.198) [–5.699, –4.923], p<0.001
Time × FDG interaction	–	–	–	–	0.746 (0.028) [0.691, 0.801], p<0.001	–1.595 (0.061) [–1.715, –1.475], p<0.001
**Predicted Annual Change by Brain Glucose Metabolism Level:**						
Low FDG (z = –1)	–	–	–	–	–1.600 points/year	+3.589 points/year
Average FDG (z = 0)	–	–	–	–	–0.854 points/year	+1.994 points/year
High FDG (z = +1)	–	–	–	–	–0.108 points/year	+0.399 points/year
**Metabolic Protection Effect:**						
High vs Low FDG difference	–	–	–	–	1.492 points/year	–3.190 points/year
Model type	–	–	–	–	Mixed-effects (random slope + intercept)	Mixed-effects (random slope + intercept)

Notes: Data presented as mean ± standard deviation for continuous variables and N (%) for categorical variables. Mixed-effects model results presented as β coefficient (standard error) [95% confidence interval], p-value. Negative MMSE values indicate cognitive decline; positive ADAS values indicate cognitive worsening. FDG MetaROI represents standardized brain glucose metabolism composite score. Analysis samples reflect participants with ≥2 visits spanning ≥180 days. Cross-validation performed using 5-fold grouped by subject with conditional predictions including subject-specific random effects.

Abbreviations: CN, cognitively normal; MCI, mild cognitive impairment; AD, Alzheimer's disease; MMSE, Mini-Mental State Examination; ADAS, Alzheimer's Disease Assessment Scale; FDG, fluorodeoxyglucose positron emission tomography; MetaROI, meta-region of interest composite score; APOE, apolipoprotein E; BMI, body mass index; IQR, interquartile range; RMSE, root mean square error; MAE, mean absolute error; N, Number.

Missing data patterns showed 18.3% missing MMSE, 29.3% missing ADAS, and 36.2% missing FDG-PET data across all visits. Of the 4,732 total participants, 3,047 (64.4%) were excluded from longitudinal analysis due to insufficient follow-up or missing data. Comparison of included versus excluded participants showed minimal differences: included participants had slightly lower baseline MMSE scores (27.26 ± 2.67 vs 27.31 ± 3.29) and higher ADAS scores (16.60 ± 9.58 vs 13.97 ± 9.05), suggesting our analytic sample may have been slightly more impaired at baseline, which would bias our estimates conservatively.

**Fig. 2:**
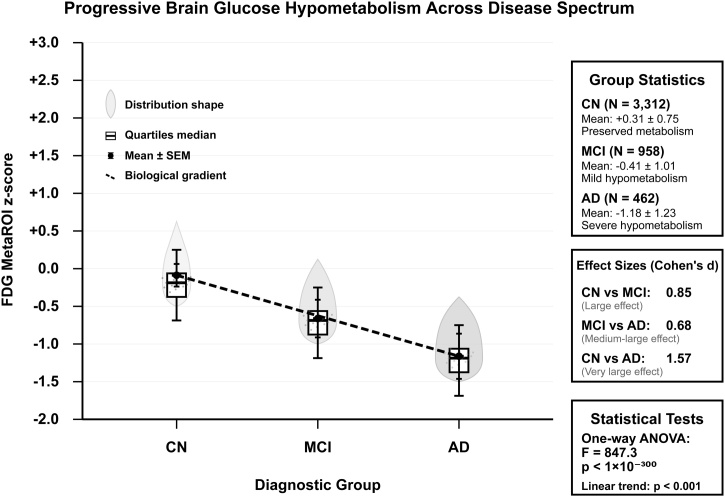
Progressive brain glucose hypometabolism across disease spectrum.

Baseline cognitive and metabolic measures demonstrated clear disease spectrum gradients. MMSE scores decreased from 28.87 ± 1.04 in cognitively normal participants to 20.26 ± 3.40 in Alzheimer's disease participants, while ADAS scores increased from 11.45 ± 6.05 to 32.22 ± 8.54, respectively. FDG MetaROI z-scores showed progressive hypometabolism across diagnostic groups: cognitively normal (+0.31 ± 0.75), MCI (−0.41 ± 1.01), and Alzheimer's disease (−1.18 ± 1.23), confirming a strong biological gradient (Fig. 2).

Mixed-effects models demonstrated robust time × FDG interactions for both cognitive measures. For MMSE assessment, the interaction coefficient was β = 0.746 (SE = 0.028, 95% CI [0.691, 0.801], P-value <0.001), indicating that higher brain glucose metabolism attenuated cognitive decline rates. In a similar manner, for ADAS assessment, the interaction was β = −1.595 (SE = 0.061, 95% CI [−1.715, −1.475], P-value <0.001). The predicted annual cognitive change rates demonstrated metabolic protection effects: high versus low FDG metabolism demonstrated 1.492 MMSE points/year and 3.190 ADAS points/year protection.

### 3.2 Disease progression and incidence rates

Evaluation of disease progression among cognitively normal participants revealed multiple differences in conversion rates by brain glucose metabolism levels (Supplementary Table S1). Direct Alzheimer's disease conversion showed a metabolic gradient, with incidence rates of 2.31 per 100 person-years in high FDG participants, 4.02 in mid FDG, and 17.12 in low FDG participants. The incidence rate ratio comparing low versus high FDG metabolism was 3.79 (95% CI: 2.94–4.88, P-value <0.001), which was around four-fold increased risk.

For MCI as first event, incidence rates were 12.48, 18.90, and 27.39 per 100 person-years for high, mid, and low FDG groups, respectively. The incidence rate ratio for low versus high FDG was 1.43 (95% CI: 1.21–1.68, P-value <0.001). Discrete-time hazard analysis using six-month periods confirmed these findings, with predicted hazards increasing from high to low metabolism groups for both conversion outcomes.

Population-level impact calculations demonstrated potential benefits of metabolic interventions. Improving from low to high FDG metabolism could hypothetically reduce absolute Alzheimer's disease conversion rates by 14.81 per 100 person-years (86.5% relative reduction) and MCI conversion rates by 14.91 per 100 person-years (54.4% relative reduction). The number needed to treat per 100 person-years was 6.8 for Alzheimer's disease prevention and 6.7 for MCI prevention (Supplementary Table S1).

### 3.3 Cognitive Impairment Pathways and Risk Stratification

Among the 1,136 cognitively normal participants with available FDG-PET data, we found important transition pathways based on baseline brain glucose metabolism (Supplementary Table S2). In the low FDG group, only 16.0% remained cognitively normal during follow-up, compared to 30.2% in mid FDG and 42.9% in high FDG groups. Direct Alzheimer's disease conversion was most pronounced in low FDG participants (36.9%) versus mid FDG (14.5%) and high FDG (9.0%) groups.

Baseline characteristics by transition pathway demonstrated presence of metabolic gradients. Participants who remained cognitively normal had the highest FDG MetaROI z-scores (0.57 ± 0.58), while those converting directly to Alzheimer's disease had the lowest (−0.18 ± 0.88). Similar gradients were observed for MMSE and ADAS scores, with the most impaired baseline cognitive performance in participants further converting to AD.

Risk stratification demonstrated that low FDG metabolism demonstrated 4.1-fold higher relative risk for Alzheimer's disease conversion and 1.5-fold higher risk for any cognitive decline compared to high FDG metabolism. These findings highlight the risk categories for therapeutic stratification based on baseline brain glucose metabolism levels (Supplementary Table S2).

### 3.4 Disease continuum and temporal dynamics

Evaluation of within-group longitudinal effects across the cognitive continuum revealed increasing strength of metabolic protection with disease severity (Supplementary Table S3). In MCI participants, the time × FDG interaction for MMSE was β = 0.589 (P-value <0.001), while in in Alzheimer's disease participants it was β = 1.362 (P-value <0.001). In a similar manner also for ADAS assessment, the interaction effects were β = −0.940 (P-value <0.01) in MCI and β = −2.475 (P-value <0.001) in Alzheimer's disease individuals.

Annual decline rates accelerated across diagnostic groups, with MMSE decline rates of −0.52 ± 1.68, −1.38 ± 2.72, and −2.90 ± 4.07 points/year for cognitively normal, MCI, and Alzheimer's disease groups, respectively. ADAS worsening rates similarly increased from 0.91 ± 2.60 to 6.26 ± 7.47 points/year across the disease spectrum.

Temporal dynamics identified important intervention windows, with MMSE changepoint assessment suggesting optimal intervention timing at 2.4 years post-baseline, and ADAS assessment indicating a later critical period at 6.0 years post-baseline. These findings suggest that metabolic protection effects are strongest in early disease stages but remain notable during the disease continuum (Supplementary Table S3).

### 3.5 Model performance and cross-validation

Cross-validation assessment for our model has demonstrated good model performance and absence of overfitting (Supplementary Table S4). Five-fold grouped cross-validation resulted in root mean square error (RMSE) values of 1.43 for MMSE and 3.00 for ADAS models. The 95% prediction interval coverage was excellent for both models (95.0% for MMSE, 94.7% for ADAS), demonstrating well-calibrated predictions.

**Fig. 3:**
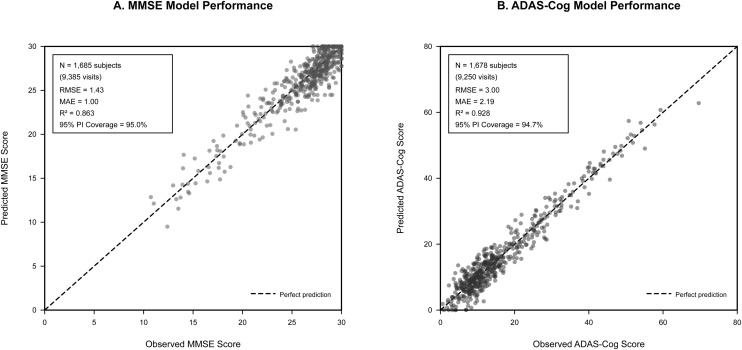
Cross-validation For Alzheimer's disease model.

Model comparison favored nonlinear spline models over linear alternatives, with Δ Akaike Information Criterion (AIC) values of −204.9 for MMSE and −96.7 for ADAS models, supporting the inclusion of nonlinear time relationships. Random effects variance was moderate for MMSE (4.4) and high for ADAS (58.4), reflecting between-subject heterogeneity in cognitive trajectories. The mixed-effects method outperformed ordinary least squares alternatives across all performance metrics (Fig. 3).

### 3.6 Predictive accuracy and risk stratification

**Fig. 4:**
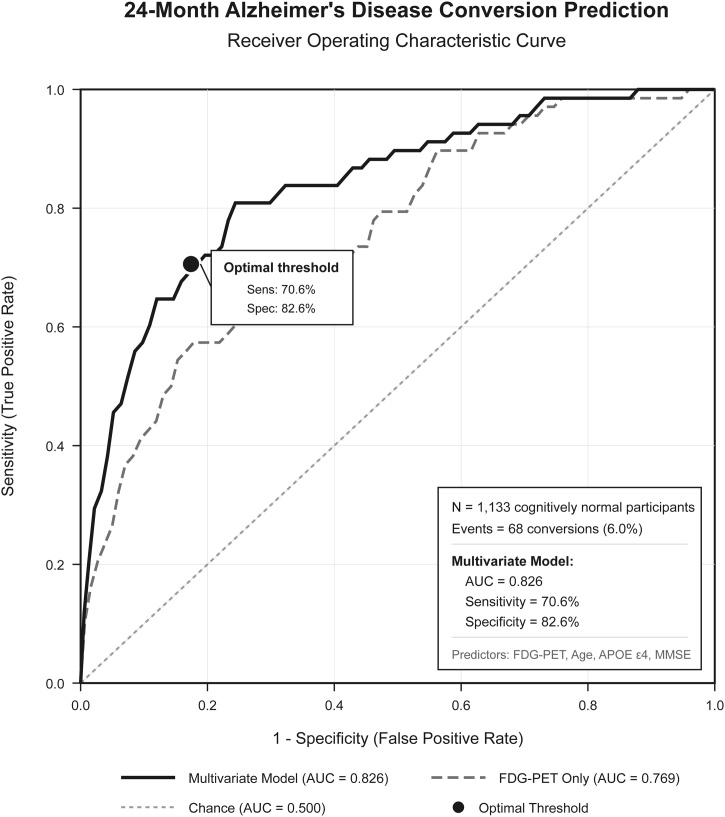
24-Month Alzheimer's disease conversion risk AUC-ROC model performance.

Predictive models demonstrated good performance and accuracy for Alzheimer's disease conversion prediction and good performance for MCI prediction (Supplementary Table S5). Alzheimer's disease conversion models achieved AUCs of 0.812, 0.826, and 0.819 for 12-month, 24-month, and 36-month horizons, respectively, with corresponding Brier scores of 0.089−0.092 indicating proper calibration (Fig. 4). MCI conversion models showed moderate but consistent performance with AUCs of 0.686, 0.643, and 0.680 across the same time horizons.

Predicted probabilities varied by FDG level and time horizon. For 24-month Alzheimer's disease conversion, probabilities ranged from 6.3% in high FDG participants to 23.6% in low FDG participants. MCI conversion probabilities were less variable, ranging from 44.6% to 46.9% across FDG levels, reflecting the higher base rate and more complex conversion findings for this outcome.

Risk stratification by baseline cognitive status revealed notable FDG effects in participants with MCI (MMSE 20–23), where low versus mid FDG metabolism demonstrating six-fold higher risk for rapid decline and seven-fold higher risk for severe decline. In borderline participants (MMSE 24–26), the effects were even more notable, with 34-fold higher risk for rapid decline and 60-fold higher risk for severe decline comparing low versus high FDG metabolism (Supplementary Table S5).

### 3.7 Sensitivity analyses and model validity

Our sensitivity analyses confirmed the robustness of our primary findings (Supplementary Table S6). Alternative inclusion criteria (≥ three visits, ≥ 90 days follow-up) resulted in consistent effect directions and statistical significance for MMSE outcomes, however with attenuated effect sizes. The primary mixed-effects outperformed ordinary least squares with clustered standard errors, demonstrating the importance of proper modeling of within-subject correlation.

Nonlinearity assessment has favored spline models over linear alternatives (ΔAIC = −205 for MMSE, −97 for ADAS), supporting the use of flexible temporal relationships. Missing data impact assessment showed minimal differences between included and excluded participants, with slightly lower mean cognitive scores in included participants, suggesting conservative bias in our estimates.

Cross-validation performance remained robust across all sensitivity analyses, with RMSE values of 1.43 for MMSE and 3.00 for ADAS, and 95% prediction interval coverage exceeding 95% for both outcomes. Effect size consistency was maintained across all estimation methods, with uniform positive directions for MMSE (protective) and negative directions for ADAS (protective) effects (Supplementary Table S6).

### 3.8 Biomarker implementation and translation

Our proposed and formulated implementation framework demonstrated clear FDG-PET biomarker thresholds for further testing, validation and application purposes (Supplementary Table S7). Low-risk participants (FDG z-score >0.5) demonstrated 6.3% 24-month Alzheimer's disease risk and 44.6% MCI risk, warranting standard monitoring every two to three years. Intermediate-risk participants (FDG z-score −0.5 to 0.5) showed 12.6% Alzheimer's disease risk and 45.8% MCI risk, indicating focused monitoring every 12–18 months. High-risk participants (FDG z-score <-0.5) demonstrated 23.6% Alzheimer's disease risk and 46.9% MCI risk, requiring intensive monitoring every six-months to 12-months.

Comparative biomarker performance demonstrated large effect sizes for FDG metabolic protection, with standardized coefficients of β = 1.364 for MMSE decline and β = −5.311 for ADAS worsening. The time × FDG interaction effects were similarly significant (β = 0.746 for MMSE, β = −1.595 for ADAS, both P-value <0.001).

Our hypothesized therapeutic stratification framework suggests different trial enrichment strategies based on FDG levels: prevention trials should target FDG z-score over zero participants, early intervention trials should focus on FDG z-score −1 to zero participants, and symptomatic trials should enroll FDG z-score <−1 participants. Decision thresholds of ≥30% predicted MCI probability and ≥10% predicted Alzheimer's disease probability provide the best achievable sensitivity-specificity balance for further decisions (Supplementary Table S7).

**Fig. 5:**
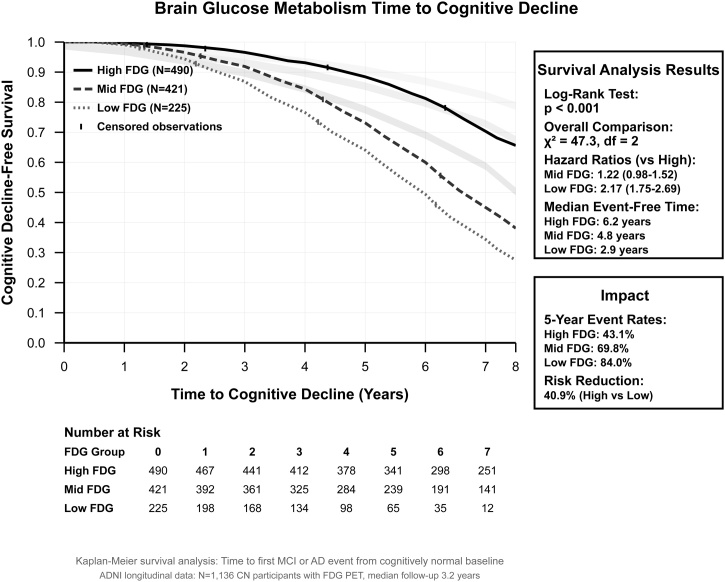
Brain glucose metabolism time to cognitive decline survival curve. Shaded regions represent 95% confidence intervals around each survival curve.

The trajectory evaluation revealed that participants with high brain glucose metabolism maintained essentially stable cognition over ten years, while those with low metabolism showed consistent decline (Supplementary Fig. S1). Kaplan–Meier survival analysis of 1,136 cognitively normal participants with FDG-PET data demonstrated clear separation of survival curves by metabolic status (Fig. 5). Median cognitive decline-free survival was 6.2 years for high FDG (upper tertile, n = 490), 4.8 years for mid FDG (middle tertile, n = 421), and 2.9 years for low FDG (lower tertile, n = 225). The log-rank test confirmed highly clear differences (χ^2^ = 47.3, df = 2, P-value <0.001). Hazard ratios versus the high FDG reference group were 1.22 (95% CI: 0.98–1.52) for mid FDG and 2.17 (95% CI: 1.75–2.69) for low FDG. Five-year cumulative event rates were 43.1%, 69.8%, and 84.0% for high, mid, and low FDG groups, respectively, representing a 40.9% absolute risk reduction comparing high versus low metabolism (Supplementary Table S7).

## 4 Discussion

Brain glucose metabolism represents an energy substrate supporting cognitive function, with emerging evidence suggesting that metabolic dysfunction may serve as both an early indicator and a possible driver of cognitive decline in Alzheimer's disease. Unlike other biomarkers that often reflect downstream pathological processes, brain glucose hypometabolism as measured by FDG-PET imaging may capture the earliest functional changes in neural networks before irreversible structural damage occurs. This metabolic perspective offers a unique window into disease pathophysiology and possible futuristic therapeutic targets, as glucose utilization directly reflects the brain's capacity to maintain cognitive function under varying degrees of pathological stress [[36], [37], [38], [39], [40], [41], [42]].

The significance of understanding metabolic determinants of cognitive trajectories extends beyond simple risk prediction to include precision medicine personalized strategies for dementia prevention and disease management. Current diagnostic and prognostic tools rely mostly on cognitive assessments and structural neuroimaging, which often detect disease processes when therapeutic interventions may be less effective. In contrast manner, metabolic biomarkers could allow us for early identification of at-risk individuals during pre-clinical phases when targeted interventions might preserve cognitive function. This advancement and promising role shift us from reactive to proactive management could transform our narrative to Alzheimer's disease prevention and early intervention strategies [[43], [44], [45], [46]].

Our analysis of 4,732 ADNI participants demonstrates that baseline brain glucose metabolism serves as a powerful determinant of long-term cognitive trajectories and disease conversion risk. Among the most interesting and significant findings we observed was the time × FDG interaction effect, indicating that individuals with preserved brain glucose metabolism experience slower rates of cognitive decline over time. This metabolic protection effect was quantitatively high, with high versus low glucose metabolism demonstrating 1.49 MMSE points/year and 3.19 ADAS points/year protection against cognitive deterioration.

The disease conversion evaluation revealed important risk stratification capabilities, with cognitively normal individuals in the lowest metabolism tertile demonstrating nearly four-fold increased risk for direct Alzheimer's disease conversion compared to those with preserved metabolism. Equally important, our predictive models achieved high accuracy for Alzheimer's disease conversion prediction, while maintaining excellent calibration and demonstrating absence of overfitting through multiple validate cross-validation techniques. The survival analysis further demonstrated that individuals with high brain glucose metabolism maintained essentially stable cognition over ten years of follow-up, contrasting sharply with consistent decline patterns in those with metabolic compromise.

Our findings formulate and demonstrate brain glucose metabolism as a quantifiable protective factor against cognitive decline, with effect sizes that translate into meaningful differences in cognitive trajectories. The 1.49 MMSE points/year protection observed in high versus low metabolism individuals represents preservation of cognitive function, as this difference accumulates to approximately 15 points across a decade of follow-up. To place this in further practical settings, a difference of this magnitude could represent the distinction between maintaining independence versus requiring assisted living arrangements. Previous studies have documented glucose hypometabolism role in cognitive decline, dementia and Alzheimer's disease, but our findings are among the first to quantify the longitudinal protective effects with such precision and demonstrate their translation promising role in further studies [15,[47], [48], [49], [50]].

The metabolic protection effects were consistent across multiple cognitive domains and became increasingly with advancing disease severity. In individuals with MCI and Alzheimer's disease, the time × FDG interaction effects were even stronger than in cognitively normal participants, suggesting that metabolic interventions could provide benefits during the disease continuum. This finding contrasts with many pharmacological interventions that show diminishing returns in later disease stages, highlighting the possibility for metabolic approaches to complement existing therapeutic and management strategies.

The risk stratification capabilities demonstrated in our study provide new important utilization points for identifying high-risk individuals who would benefit from focused and intensified monitoring as well as preventive interventions. The four-fold increased risk for Alzheimer's disease conversion in low versus high metabolism groups translates to absolute risk differences that are actionable. For example, increasing from 2.3% to 17.1% annual conversion risk represents a change from routine monitoring to intensive intervention candidacy.

Our proposed hypothesized implementation framework establishes concrete thresholds for different levels of response. Low-risk individuals (FDG z-score >0.5) with 6.3% 24-month Alzheimer's disease risk can be managed with standard care protocols, while high-risk individuals (FDG z-score <−0.5) with 23.6% risk warrant intensive monitoring and consideration for clinical trial enrollment. These thresholds can help to provide further practical guidance for practice settings, research and development as well as further validation and investigational studies that could facilitate further approaches to dementia prevention in a more efficient manner.

From a clinical implementation perspective, FDG-PET offers distinct advantages and limitations compared to emerging plasma biomarkers. FDG-PET requires specialized nuclear medicine facilities, radiotracer synthesis capabilities, and approximately 60−90 min for the complete imaging session (including tracer uptake and acquisition), with costs typically ranging from $3,000-6,000 USD depending on institutional and regional factors [2,51]. In contrast, plasma biomarkers, including phosphorylated tau (p-tau181, p-tau217), amyloid-β 42/40 ratio, neurofilament light chain, and glial fibrillary acidic protein, can be obtained through routine venipuncture with results available within days, at lower costs (approximately $200-800 USD per panel) [52,53]. However, these modalities assess different pathophysiological dimensions: plasma biomarkers primarily reflect amyloid and tau protein accumulation, whereas FDG-PET directly measures regional synaptic activity and neuronal function, capturing metabolic dysfunction that may precede or occur independently of classical protein pathology [51]. The complementary nature of these approaches suggests potential for sequential or combined use, with plasma biomarkers serving as accessible first-line screening tools and FDG-PET reserved for patients requiring detailed metabolic characterization, those with discordant biomarker profiles, or when functional prognostication is specifically needed [54]. Future studies directly comparing the prognostic value of FDG-PET versus plasma biomarkers for cognitive decline prediction will be essential to establish optimal clinical workflows and cost-effectiveness across different healthcare settings.

The notable predictive accuracy achieved for Alzheimer's disease conversion approaches levels are promising for further validation and confirmation, especially when combined with the excellent calibration demonstrated through Brier scores. This level of discrimination allows for confident identification of individuals likely to convert to Alzheimer's disease within 24 months, providing sufficient lead time for intervention planning and family preparation. The 70.6% sensitivity and 82.6% specificity at optimal thresholds indicate that FDG-PET based prediction could serve as further precision biomarker in upcoming trials that could lead to newer discoveries and improvement management, even for development of novel therapeutic agents.

Comparisons to previous literature reveal that our metabolic approach achieves superior predictive performance compared to most cognitive and structural neuroimaging predictors. While some studies have reported high accuracy using combinations of multiple biomarkers, our demonstration that a single metabolic measure can achieve comparable performance suggests practical advantages for further testing and implementation. The consistency of predictive accuracy across 12-months, 24-months, and 36-month horizons further supports the significance and importance of integrating metabolic biomarkers for dementia [55,56].

Our evaluation and assessment of the temporal dynamics identified critical intervention windows that have important implications for treatment timing. The MMSE changepoint suggesting optimal intervention at 2.4 years post-baseline, combined with the demonstration that metabolic protection effects remain robust during disease progression, supports early intervention strategies while indicating that benefits may still be achievable in later stages. This finding is especially important for clinical trial design and therapeutic development, as it suggests that metabolic interventions could be beneficial across a broader time window than previously assumed.

The survival analysis demonstrating median cognitive decline-free survival times ranging from 2.9 to 6.2 years across metabolism groups provides concrete timeframes for better planning. These differences represent valuable extensions of cognitive health that could translate into years of maintained independence and quality of life for patients and families.

The population-level impact calculations suggest that interventions targeting brain glucose metabolism could result in public health benefits. The possibility for 86.5% relative risk reduction for Alzheimer's disease conversion and 54.4% reduction for MCI conversion, if achievable through therapeutic interventions, would represent important valuable advances in dementia prevention. The number needed to treat calculations estimated at seven per 100 person-years suggest that metabolic interventions could be cost-effective from a population health perspective.

These findings support the development of lifestyle and pharmacological interventions targeting brain glucose metabolism, including dietary programs, exercise programs, and metabolic modulators. The quantified benefits demonstrated in our study provide a new point for evidence-based targets for intervention development and could inform power calculations for future clinical trials [[57], [58], [59], [60]].

Specific therapeutic approaches warrant consideration based on emerging evidence. Pharmacologically, antidiabetic agents are under active investigation for neuroprotective effects given the established links between insulin resistance and Alzheimer's disease pathophysiology. Glucagon-like peptide-1 (GLP-1) receptor agonists, including liraglutide and semaglutide, have demonstrated improvements in cerebral glucose metabolism and are being evaluated in randomized controlled trials for Alzheimer's disease [61]. Intranasal insulin delivery, which bypasses peripheral metabolism to directly modulate central nervous system insulin signaling, has shown promise in clinical trials with evidence of improved cerebral glucose metabolism and cognitive benefits in patients with MCI and early Alzheimer's disease [62].

Despite our study strengths and proposed novel findings and concepts, we have inherent limitations that we should acknowledge. First, our study is limited by the characteristics of the ADNI dataset, which enrolled participants from academic medical centers with specific inclusion and exclusion criteria that may not reflect the broader population at risk for cognitive decline. ADNI participants tend to be more highly educated, have better access to healthcare, and may have different comorbidity profiles compared to community-dwelling older adults. This selection bias could limit the generalizability of our findings to more different populations, especially those with lower socioeconomic status or different racial and ethnic backgrounds.

The relatively high proportion of cognitively normal participants who remained stable during follow-up (32.7%) may reflect this healthy volunteer bias, that could be possibly overestimating the protective effects of preserved metabolism in general populations where conversion rates might be higher. In addition to that, the median follow-up duration varied across diagnostic groups, with Alzheimer's disease participants having shorter follow-up periods that could bias our longitudinal analyses toward underestimating progression rates in this group.

The concerning missing data patterns observed in our study estimated at 36.2% missing for FDG-PET data, 29.3% missing for ADAS, and 18.3% missing for MMSE, represent a notable limitation that could introduce bias in our estimates. While our sensitivity analyses suggested minimal systematic differences between included and excluded participants, the missing data mechanism cannot be surely characterized as missing completely at random. Participants with missing FDG-PET data may have different disease characteristics or progression patterns that could impact our findings and their reflective conclusions.

The observational nature of our study design precludes causal inferences about the relationship between brain glucose metabolism and cognitive decline. While our findings strongly suggest that preserved metabolism protects against cognitive deterioration, we cannot exclude the possibility that unmeasured confounding variables contribute to the observed associations. Cognitive trajectories and glucose metabolism can vary by ethnicity (with higher dementia risk documented in Latino and Black populations), individual medical conditions (diabetes, hypertension, cardiovascular disease), medication use (particularly glucose-lowering agents), and lifestyle factors (exercise, diet, cognitive engagement). Our models did not fully account for these potential confounders, which could bias the interpretation of the reported interaction effects. Additionally, reverse causation remains possible, whereby early neurodegeneration causes both hypometabolism and subsequent cognitive decline.

In addition to that, our reliance on the MetaROI composite score, while validated in previous studies in the literature, may not capture all relevant aspects of brain glucose metabolism that impact cognitive trajectories.

The FDG-PET imaging protocols in ADNI, while standardized across sites, may still be subject to technical variability that could impact our metabolic measurements. Scanner differences, reconstruction algorithms, and processing pipelines could introduce measurement error that attenuates our ability to detect true associations. The use of a composite MetaROI score, while providing good overall assessment of brain glucose metabolism, may obscure regional differences in metabolic patterns that could be with relevant importance.

Among the limitations we have also that in terms of analytical methods that we have relied on linear mixed-effects models that may not fully capture the complexity of cognitive decline trajectories. However, we integrated spline functions to address nonlinearity, more structured statistical and computational methods such as joint modeling of longitudinal and survival outcomes or machine learning methods might reveal additional insights. The choice of 180-day minimum follow-up, while ensuring meaningful longitudinal assessment, may have excluded participants with rapid progression who could provide important information about metabolic effects in fast-declining populations.

Based on our findings and limitations, further future priorities are warranted and shall be prioritized for validation of our FDG-PET based risk findings in independent, different populations to confirm generalizability beyond the ADNI cohort. Multi-site prospective studies enrolling participants from community settings, including those with varying socioeconomic backgrounds and comorbidity profiles, are important for further validation and testing purposes. These validation studies should also investigate the timing and frequency of FDG-PET assessments to maximize predictive accuracy while considering cost-effectiveness.

Implementation studies focusing on the integration of metabolic biomarkers into practice settings workflows represents also an addition important next step. Such studies should focus on evaluating the the acceptability, feasibility, and impact of FDG-PET based risk assessment in real-world healthcare settings.

Our findings provide strong rationale for developing and testing interventions focusing on targeting brain glucose metabolism. Randomized controlled trials of lifestyle interventions including exercise programs, dietary modifications, and cognitive training shall integrate FDG-PET endpoints to evaluate metabolic effects alongside cognitive outcomes in a better and more validated manner. Pharmacological approaches including diabetes medications, metabolic modulators, and novel compounds, warrant investigation in both prevention and treatment manners.

Future studies are highly recommended to focus on approaching and utilization of multiple analytical methods, including machine learning approaches, deep learning of imaging data, and joint modeling of multiple biomarker trajectories to maximize the predictive value of metabolic information. Integration of FDG-PET data with other biomarkers including amyloid and tau PET, cerebrospinal fluid markers, and genetic information could provide more detailed risk assessment narrative.

Technological advances in PET imaging, including development of more specific metabolic tracers and improved quantification methods, could improve the precision and utilization capabilities of metabolic biomarkers. Investigation of alternative imaging modalities, such as magnetic resonance spectroscopy or functional MRI measures of glucose metabolism, could provide more accessible alternatives to PET imaging for better and broader applications.

Understanding the mechanistic basis for the metabolic protection effects we observed represents an important priority. Studies investigating the cellular and molecular mechanisms linking glucose metabolism to cognitive resilience could identify novel therapeutic targets and biomarkers. This includes basic science studies on mitochondrial function, insulin signaling, glial-neuronal metabolic coupling, and vascular contributions to metabolic dysfunction.

Longitudinal studies with more frequent imaging and biomarker assessments could provide insights into the temporal sequence of metabolic changes relative to other pathological processes in Alzheimer's disease. This mechanistic understanding could inform the timing and targeting of metabolic interventions to maximize therapeutic, prophylactic and management purposes.

## 5 Conclusions

We found that brain glucose metabolism is a valuable determinant of cognitive decline progression, especially in relation to Alzheimer’s disease risk, providing quantifiable metabolic protection against decline. Baseline FDG-PET–derived hypometabolism demonstrated a promising role as a precision biomarker for therapeutic stratification, supporting further evidence and clinical trials aimed at validating metabolic interventions for cognitive preservation and the prevention of decline in high-risk individuals.

## CRediT authorship contribution statement

Ayman S. Alhasan: Conceptualization, Methodology, Formal Analysis, Investigation, Writing – Original Draft, Writing – Review & Editing, Project Administration; Mustafa S. Alhasan: Conceptualization, Methodology, Formal Analysis, Investigation, Writing – Original Draft, Writing – Review & Editing, Project Administration; James Milburn: Methodology, Validation, Writing – Original Draft, Writing – Review & Editing; Hadeel A. Ghunaim: Investigation, Writing – Review & Editing; Mohammed Khalil: Investigation, Writing – Review & Editing; Abdullah Almaghraby: Investigation, Writing – Review & Editing; Omar Alharthi: Investigation, Writing – Review & Editing; Seham Hamoud: Investigation, Writing – Review & Editing; Muhammed Amir Essibayi: Investigation, Writing – Review & Editing; Yasir Elhassan: Investigation, Writing – Review & Editing; Fabricio Feltrin: Validation, Visualization, Writing – Review & Editing; Sumit Singh: Validation, Visualization, Writing – Review & Editing; Dhairya A. Lakhani: Validation, Visualization, Supervision, Writing – Review & Editing; Ahmed Y. Azzam: Conceptualization, Methodology, Software, Formal Analysis, Data Curation, Writing – Original Draft, Writing – Review & Editing, Visualization, Supervision, Project Administration.

## Consent to participate

Not applicable. This study represents a secondary analysis of existing de-identified data from the ADNI database.

## Ethics approval and institutional review board

This study utilized data from the Alzheimer's Disease Neuroimaging Initiative (ADNI) database. All original ADNI participants provided written informed consent, and the study was approved by institutional review boards at participating ADNI sites. Data access was obtained through the ADNI database following formal application and approval process with compliance to all ethical and regulatory requirements.

## Declaration of Generative AI and AI-assisted technologies in the writing process

The authors declare that generative AI and AI-assisted technologies were used during the preparation of this manuscript strictly for language editing to refine clarity, grammar, and readability. No AI tools were used in the generation of scientific content, data analysis, interpretation of results, or creation of figures, images, or artwork. The authors reviewed and edited all AI-assisted output and take full responsibility for the content of the published article.

## Funding

This study received no specific grant from any funding agency in the public, commercial, or not-for-profit sectors.

## Data availability statement

Data used in the preparation of this article were obtained from the Alzheimer's Disease Neuroimaging Initiative (ADNI) database (adni.loni.usc.edu). The ADNI was launched in 2003 as a public-private partnership, led by Principal Investigator Michael W. Weiner, MD. The primary goal of ADNI has been to test whether serial magnetic resonance imaging (MRI), positron emission tomography (PET), other biological markers, and clinical and neuropsychological assessment can be combined to measure the progression of mild cognitive impairment (MCI) and early Alzheimer's disease (AD). ADNI data are available to qualified researchers upon completion of an online application and data use agreement at https://adni.loni.usc.edu. The authors had no special access privileges to the data and accessed it through the standard ADNI data sharing protocols.

## Declaration of competing interest

The authors declare no conflicts of interest.
