# Supplementary material for: Baseline FDG-PET Brain hypometabolism as a predictive biomarker of cognitive decline and Alzheimer’s disease risk
Source: J Nutr Health Aging. 2026 Mar 11;30(5):100823. doi: 10.1016/j.jnha.2026.100823 (PMC12994019; doi:10.1016/j.jnha.2026.100823)
Supplement: Supplementary file 1 [file mmc1.docx]

**Supplementary Table 1:** Incidence Rate and Disease Progression.

| **Metric** | **High FDG** | **Mid FDG** | **Low FDG** | **Overall** |
| --- | --- | --- | --- | --- |
| **AD Direct Conversion from CN:** | | | | |
| Events, N | 31 | 42 | 115 | 188 |
| Person-years | 1,339.9 | 1,046.0 | 671.7 | 3,057.6 |
| Incidence rate per 100 PY | 2.31 | 4.02 | 17.12 | 6.15 |
| Incidence rate ratio (95% CI) | Reference | 0.89 (0.63–1.26) | 3.79 (2.94–4.88) | — |
| P-value | — | 0.528 | <0.001 | — |
| **MCI as First Event from CN:** | | | | |
| Events, N | 175 | 208 | 191 | 574 |
| Person-years | 1,401.8 | 1,100.8 | 697.3 | 3,199.9 |
| Incidence rate per 100 PY | 12.48 | 18.90 | 27.39 | 17.94 |
| Incidence rate ratio (95% CI) | Reference | 0.98 (0.84–1.15) | 1.43 (1.21–1.68) | — |
| P-value | — | 0.825 | <0.001 | — |
| **Discrete-Time Hazard Analysis (6-month periods):** | | | | |
| **AD direct conversion:** | | | | |
| FDG hazard ratio per SD | — | — | — | 0.97 |
| **Predicted 6-month hazard by FDG z-score:** | | | | |
| FDG z = +1 (High) | 0.300 | — | — | — |
| FDG z = 0 (Average) | — | 0.309 | — | — |
| FDG z = -1 (Low) | — | — | 0.319 | — |
| **MCI as first event:** | | | | |
| FDG hazard ratio per SD | — | — | — | 0.97 |
| **Predicted 6-month hazard by FDG z-score:** | | | | |
| FDG z = +1 (High) | 0.311 | — | — | — |
| FDG z = 0 (Average) | — | 0.320 | — | — |
| FDG z = -1 (Low) | — | — | 0.328 | — |
| **Population-Level Impact of Metabolic Interventions:** | | | | |
| AD direct conversion: |  |  |  |  |
| Absolute rate reduction (Low→High FDG) | — | — | 14.81 per 100 PY | — |
| Relative rate reduction (Low→High FDG) | — | — | 86.5% | — |
| Number needed to treat per 100 PY | — | — | 6.8 | — |
| **MCI as first event:** | | | | |
| Absolute rate reduction (Low→High FDG) | — | — | 14.91 per 100 PY | — |
| Relative rate reduction (Low→High FDG) | — | — | 54.4% | — |
| Number needed to treat per 100 PY | — | — | 6.7 | — |
| **Risk Stratification:** | | | | |
| AD conversion risk category | Low risk | Intermediate risk | High risk | — |
| Risk level vs High FDG | Baseline | 1.7× higher | 7.4× higher | — |
| MCI conversion risk category | Low risk | Intermediate risk | High risk | — |
| Risk level vs High FDG | Baseline | 1.5× higher | 2.2× higher | — |

***Abbreviations:*** *CN, cognitively normal; MCI, mild cognitive impairment; AD, Alzheimer's disease; FDG, fluorodeoxyglucose positron emission tomography; PY, person-years; CI, confidence interval; SD, standard deviation; N, Number.*
