# Supplementary material for: Baseline FDG-PET Brain hypometabolism as a predictive biomarker of cognitive decline and Alzheimer’s disease risk
Source: J Nutr Health Aging. 2026 Mar 11;30(5):100823. doi: 10.1016/j.jnha.2026.100823 (PMC12994019; doi:10.1016/j.jnha.2026.100823)
Supplement: Supplementary file 2 [file mmc2.docx]

**Supplementary Table 2:** Cognitive Impairment and Disease Conversion Risk from Normal Cognition.

| **Characteristic** | **Low FDG (N=227)** | **Mid FDG (N=423)** | **High FDG (N=489)** | **Overall (N=1,139)** |
| --- | --- | --- | --- | --- |
| **Transition Pathways from CN, N (%):** | | | | |
| Remained CN | 36 (15.9) | 127 (30.0) | 210 (42.9) | 373 (32.7) |
| CN → MCI only | 50 (22.0) | 162 (38.3) | 184 (37.6) | 396 (34.8) |
| CN → MCI → AD | 56 (24.7) | 71 (16.8) | 52 (10.6) | 179 (15.7) |
| CN → AD (direct) | 85 (37.4) | 63 (14.9) | 43 (8.8) | 191 (16.8) |
| Any cognitive decline | 191 (84.1) | 296 (70.0) | 279 (57.1) | 766 (67.3) |
| **Baseline Characteristics by Transition Pathway, Mean ± SD:** | | | | |
| **Remained CN:** | | | | |
| FDG MetaROI z-score | — | — | — | 0.57 ± 0.58 |
| MMSE score | — | — | — | 28.99 ± 1.00 |
| ADAS score | — | — | — | 6.34 ± 2.39 |
| **CN → MCI only:** | | | | |
| FDG MetaROI z-score | — | — | — | 0.43 ± 0.65 |
| MMSE score | — | — | — | 28.88 ± 1.03 |
| ADAS score | — | — | — | 12.58 ± 3.15 |
| **CN → MCI → AD:** | | | | |
| FDG MetaROI z-score | — | — | — | 0.04 ± 0.78 |
| MMSE score | — | — | — | 28.48 ± 1.03 |
| ADAS score | — | — | — | 14.30 ± 3.28 |
| **CN → AD (direct):** | | | | |
| FDG MetaROI z-score | — | — | — | -0.18 ± 0.88 |
| MMSE score | — | — | — | 28.19 ± 1.01 |
| ADAS score | — | — | — | 21.52 ± 5.41 |
| **Incidence Rates per 100 Person-Years:** | | | | |
| **MCI as first event:** | | | | |
| Events/Person-years | 191/697 | 208/1,101 | 175/1,402 | 574/3,200 |
| Rate per 100 PY | 27.39 | 18.90 | 12.48 | 17.94 |
| **AD direct conversion:** | | | | |
| Events/Person-years | 115/672 | 42/1,046 | 31/1,340 | 188/3,058 |
| Rate per 100 PY | 17.12 | 4.02 | 2.31 | 6.15 |
| **Incidence Rate Ratios (95% CI):** | | | | |
| MCI as first event vs High FDG | 1.43 (1.21–1.68) | 0.98 (0.84–1.15) | Reference | — |
| AD direct conversion vs High FDG | 3.79 (2.94–4.88) | 0.89 (0.63–1.26) | Reference | — |
| **Risk Stratification:** | | | | |
| Relative risk for AD conversion | 4.1× higher | 1.0× (similar) | Reference | — |
| Relative risk for any decline | 1.5× higher | 1.2× higher | Reference | — |

***Note:*** *Data represent cognitively normal participants at baseline with available FDG PET data (N=1,139 of 3,312 total CN participants). FDG groups defined by tertiles of baseline brain glucose metabolism. Transition pathways determined by longitudinal follow-up. Incidence rates calculated using Poisson regression. Rate ratios compare Low and Mid FDG groups to High FDG (reference group). Abbreviations: CN, cognitively normal; MCI, mild cognitive impairment; AD, Alzheimer's disease; FDG, fluorodeoxyglucose positron emission tomography; MMSE, Mini-Mental State Examination; ADAS, Alzheimer's Disease Assessment Scale; MetaROI, meta-region of interest composite score; PY, person-years; CI, confidence interval; N, Number.*
