# Supplementary material for: Baseline FDG-PET Brain hypometabolism as a predictive biomarker of cognitive decline and Alzheimer’s disease risk
Source: J Nutr Health Aging. 2026 Mar 11;30(5):100823. doi: 10.1016/j.jnha.2026.100823 (PMC12994019; doi:10.1016/j.jnha.2026.100823)
Supplement: Supplementary file 3 [file mmc3.docx]

**Supplementary Table 3:** Cognitive Continuum Characteristics, Within-Group Effects, and Temporal Dynamics.

| **Characteristic** | **CN** | **MCI** | **AD** | **Gradient Direction** |
| --- | --- | --- | --- | --- |
| **Baseline Characteristics (Biological Gradient):** | | | | |
| Participants, N | 3,312 | 958 | 462 | — |
| MMSE score, mean ± SD | 28.87 ± 1.04 | 25.21 ± 0.88 | 20.26 ± 3.40 | Declining |
| ADAS score, mean ± SD | 11.45 ± 6.05 | 21.72 ± 7.60 | 32.22 ± 8.54 | Worsening |
| FDG MetaROI z-score, mean ± SD | 0.31 ± 0.75 | -0.41 ± 1.01 | -1.18 ± 1.23 | Hypometabolism |
| Follow-up, median (IQR), years | 0.25 (0.00, 4.20) | 0.60 (0.00, 2.24) | 0.12 (0.00, 2.02) | Shorter follow-up |
| **Within-Group Longitudinal Effects (Time × FDG Interaction):** | | | | |
| **MMSE assessment:** | | | | |
| Participants, N | 1,136 | 379 | 170 | — |
| Visits, N | 7,178 | 1,707 | 500 | — |
| Time effect, β (SE) | — | -0.613 (0.102) | -1.949 (0.310) | Faster decline |
| FDG effect, β (SE) | — | 1.842 (0.178) | 1.396 (0.296) | Metabolic protection |
| Time × FDG, β (SE) | — | 0.589 (0.098) | 1.362 (0.181) | Stronger effect |
| Time × FDG, p-value | — | <0.001 | <0.001 | — |
| **ADAS assessment:** | | | | |
| Participants, N | 1,133 | 376 | 169 | — |
| Visits, N | 7,106 | 1,667 | 477 | — |
| Time effect, β (SE) | — | 1.014 (0.291) | 3.155 (0.731) | Faster worsening |
| FDG effect, β (SE) | — | -5.307 (0.529) | -4.488 (0.873) | Metabolic protection |
| Time × FDG, β (SE) | — | -0.940 (0.293) | -2.475 (0.412) | Stronger effect |
| Time × FDG, p-value | — | <0.01 | <0.001 | — |
| **Annual Decline Rates (per-subject slopes), Mean ± SD:** | | | | |
| MMSE decline, points/year | -0.52 ± 1.68 | -1.38 ± 2.72 | -2.90 ± 4.07 | Accelerating decline |
| ADAS worsening, points/year | 0.91 ± 2.60 | 3.66 ± 6.78 | 6.26 ± 7.47 | Accelerating worsening |
| **Temporal Dynamics (Critical Intervention Windows):** | | | | |
| **MMSE changepoint assessment:** | | | | |
| Critical period, years | — | — | — | 2.4 years post-baseline |
| Pre-period slope | — | — | — | -0.516 points/year |
| Post-period slope | — | — | — | -0.505 points/year |
| Pre-period Time×FDG effect | — | — | — | 0.566 |
| Post-period Time×FDG effect | — | — | — | 0.468 |
| **ADAS changepoint assessment:** | | | | |
| Critical period, years | — | — | — | 6.0 years post-baseline |
| Pre-period slope | — | — | — | 0.462 points/year |
| Post-period slope | — | — | — | -0.067 points/year |
| Pre-period Time×FDG effect | — | — | — | -0.509 |
| Post-period Time×FDG effect | — | — | — | 0.576 |
| **Implications:** | | | | |
| Metabolic protection strength | Baseline effect | Moderate effect | Strong effect | Increasing |
| Therapeutic window priority | Prevention | Early intervention | Symptomatic treatment | — |
| Best intervention timing | Preclinical | Early MCI | Early-moderate AD | — |

***Abbreviations:*** *CN, cognitively normal; MCI, mild cognitive impairment; AD, Alzheimer's disease; MMSE, Mini-Mental State Examination; ADAS, Alzheimer's Disease Assessment Scale; FDG, fluorodeoxyglucose positron emission tomography; MetaROI, meta-region of interest composite score; SE, standard error; IQR, interquartile range; N, Number.*
