# Supplementary material for: Baseline FDG-PET Brain hypometabolism as a predictive biomarker of cognitive decline and Alzheimer’s disease risk
Source: J Nutr Health Aging. 2026 Mar 11;30(5):100823. doi: 10.1016/j.jnha.2026.100823 (PMC12994019; doi:10.1016/j.jnha.2026.100823)
Supplement: Supplementary file 4 [file mmc4.docx]

**Supplementary Table 4:** Cross-Validation Performance of Predictive Models.

| **Performance Metric** | **MMSE Assessment** | **ADAS Assessment** |
| --- | --- | --- |
| **Cross-Validation Performance (5-Fold Grouped by Subject):** | | |
| Root mean square error (RMSE) | 1.43 | 3.00 |
| Mean absolute error (MAE) | 1.00 | 2.19 |
| Conditional R² | 0.863 | 0.928 |
| 95% prediction interval coverage, % | 95.0 | 94.7 |
| **Model Comparison and Selection:** | | |
| **Mixed-effects model (primary):** | | |
| Random effects variance | 4.439 | 58.388 |
| Subjects, N | 1,685 | 1,678 |
| Visits, N | 9,385 | 9,250 |
| **OLS model (sensitivity analysis):** | | |
| Akaike Information Criterion (AIC) | 49,471.5 | 67,996.2 |
| Bayesian Information Criterion (BIC) | 49,500.1 | 68,024.7 |
| Subjects, N | 1,685 | 1,678 |
| Visits, N | 9,385 | 9,250 |
| **Nonlinearity Assessment:** | | |
| Linear model AIC | 49,471.5 | 67,996.2 |
| Spline model AIC | 49,266.6 | 67,899.5 |
| ΔAIC (Spline - Linear) | -204.9 | -96.7 |
| Linear model BIC | 49,500.1 | 68,024.7 |
| Spline model BIC | 49,338.1 | 67,970.8 |
| ΔBIC (Spline - Linear) | -161.9 | -53.9 |
| **Model Quality Assessment:** | | |
| Calibration quality | Excellent (95.0% coverage) | Excellent (94.7% coverage) |
| Predictive accuracy | Excellent (R² = 0.863) | Excellent (R² = 0.928) |
| Nonlinear relationships | Supported (ΔAIC = -204.9) | Supported (ΔAIC = -96.7) |
| Between-subject heterogeneity | Moderate (RE variance = 4.4) | High (RE variance = 58.4) |
| Model significance | High (consistent across methods) | High (consistent across methods) |

***Notes:*** *Cross-validation performed using 5-fold methodology with subjects grouped to maintain independence. Conditional predictions including subject-specific random effects were used for model evaluation, which is standard practice for mixed-effects models. Prediction intervals calculated using mixed-effects model predictions. Lower AIC/BIC values indicate better model fit. Negative ΔAIC/ΔBIC values favor the spline (nonlinear) model. Random effects variance quantifies between-subject variability in cognitive trajectories. Abbreviations: MMSE, Mini-Mental State Examination; ADAS, Alzheimer's Disease Assessment Scale; OLS, ordinary least squares; AIC, Akaike Information Criterion; BIC, Bayesian Information Criterion; RE, random effects; RMSE, root mean square error; MAE, mean absolute error; N, Number.*
