# Supplementary material for: Baseline FDG-PET Brain hypometabolism as a predictive biomarker of cognitive decline and Alzheimer’s disease risk
Source: J Nutr Health Aging. 2026 Mar 11;30(5):100823. doi: 10.1016/j.jnha.2026.100823 (PMC12994019; doi:10.1016/j.jnha.2026.100823)
Supplement: Supplementary file 5 [file mmc5.docx]

**Supplementary Table 5:** Predictive Accuracy and Risk Stratification.

| **Metric** | **12 Months** | **24 Months** | **36 Months** | **FDG Level** | **Risk Outcomes** |
| --- | --- | --- | --- | --- | --- |
| **Predictive Model Performance (N=1,133):** | | | | | |
| **MCI conversion prediction:** | | | | | |
| Area under curve (AUC) | 0.686 | 0.643 | 0.680 | — | — |
| Brier score | 0.239 | 0.244 | 0.241 | — | — |
| Events, N (%) | 514 (45.4) | 520 (45.9) | 525 (46.3) | — | — |
| **AD conversion prediction:** | | | | | |
| Area under curve (AUC) | 0.812 | 0.826 | 0.819 | — | — |
| Brier score | 0.089 | 0.092 | 0.091 | — | — |
| Events, N (%) | 167 (14.7) | 168 (14.8) | 168 (14.8) | — | — |
| **Predicted Probabilities by FDG Level, %:** | | | | | |
| MCI conversion | 46.5 | 46.9 | 47.4 | Low FDG (z = -1) | — |
| MCI conversion | 45.3 | 45.8 | 46.2 | Average FDG (z = 0) | — |
| MCI conversion | 44.0 | 44.6 | 45.1 | High FDG (z = +1) | — |
| AD conversion | 23.5 | 23.6 | 23.6 | Low FDG (z = -1) | — |
| AD conversion | 12.5 | 12.6 | 12.6 | Average FDG (z = 0) | — |
| AD conversion | 6.2 | 6.3 | 6.3 | High FDG (z = +1) | — |
| Remain CN | 30.0 | 29.5 | 29.0 | Low FDG (z = -1) | — |
| Remain CN | 42.2 | 41.6 | 41.2 | Average FDG (z = 0) | — |
| Remain CN | 49.8 | 49.1 | 48.6 | High FDG (z = +1) | — |
| **Risk Stratification by Baseline Status:** | | | | | |
| **MMSE 20–23 (mild impairment):** | | | | | |
| Rapid decline (≥3 points), % | — | 2.0 | 3.4 | Mid FDG | Risk ratio: ~6× higher (Low vs Mid) |
| Rapid decline (≥3 points), % | — | 17.2 | 20.1 | Low FDG | Risk ratio: ~6× higher (Low vs Mid) |
| Severe decline (≥5 points), % | — | 1.1 | 2.0 | Mid FDG | Risk ratio: ~7× higher (Low vs Mid) |
| Severe decline (≥5 points), % | — | 9.6 | 13.6 | Low FDG | Risk ratio: ~7× higher (Low vs Mid) |
| **MMSE 24–26 (borderline):** | | | | | |
| Rapid decline (≥3 points), % | — | 0.3 | 0.3 | High FDG | Risk ratio: ~34× higher (Low vs High) |
| Rapid decline (≥3 points), % | — | 3.2 | 3.9 | Mid FDG | Risk ratio: ~34× higher (Low vs High) |
| Rapid decline (≥3 points), % | — | 8.3 | 10.3 | Low FDG | Risk ratio: ~34× higher (Low vs High) |
| Severe decline (≥5 points), % | — | 0.0 | 0.0 | High FDG | Low FDG shows 60× higher risk |
| Severe decline (≥5 points), % | — | 0.9 | 1.1 | Mid FDG | Low FDG shows 60× higher risk |
| Severe decline (≥5 points), % | — | 4.0 | 6.0 | Low FDG | Low FDG shows 60× higher risk |
| **MMSE ≥27 (normal):** | | | | | |
| Rapid decline (≥3 points), % | — | 1.8 | 2.4 | High FDG | Minimal FDG effect in normal range |
| Rapid decline (≥3 points), % | — | 2.0 | 2.9 | Mid FDG | Minimal FDG effect in normal range |
| Rapid decline (≥3 points), % | — | 1.9 | 2.6 | Low FDG | Minimal FDG effect in normal range |
| **ADAS ≥30 (severe impairment):** | | | | | |
| Severe worsening (≥5 points), % | — | 1.4 | 2.0 | High FDG | Risk ratio: ~11× higher (Low vs High) |
| Severe worsening (≥5 points), % | — | 3.9 | 5.0 | Mid FDG | Risk ratio: ~11× higher (Low vs High) |
| Severe worsening (≥5 points), % | — | 18.8 | 22.4 | Low FDG | Risk ratio: ~11× higher (Low vs High) |

***Notes:*** *Predictive models trained on cognitively normal participants at baseline using 5-fold stratified cross-validation. AUC values >0.7 indicate acceptable discrimination; >0.8 good discrimination. Lower Brier scores indicate better calibration. Clinical risk stratification based on baseline cognitive status and FDG tertiles. Risk ratios compare low to high brain glucose metabolism groups. Abbreviations: MCI, mild cognitive impairment; AD, Alzheimer's disease; CN, cognitively normal; FDG, fluorodeoxyglucose positron emission tomography; MMSE, Mini-Mental State Examination; ADAS, Alzheimer's Disease Assessment Scale; AUC, area under curve; N, Number.*
