# Supplementary material for: Baseline FDG-PET Brain hypometabolism as a predictive biomarker of cognitive decline and Alzheimer’s disease risk
Source: J Nutr Health Aging. 2026 Mar 11;30(5):100823. doi: 10.1016/j.jnha.2026.100823 (PMC12994019; doi:10.1016/j.jnha.2026.100823)
Supplement: Supplementary file 6 [file mmc6.docx]

**Supplementary Table 6:** Sensitivity Analyses and Model Validity.

| **Analysis Type** | **Specification** | **MMSE Results** | **ADAS Results** | **Interpretation** |
| --- | --- | --- | --- | --- |
| **Primary vs Alternative Estimators:** | | | | |
| Mixed-effects model (primary) | Random slope + intercept | — | — | — |
| Time × FDG interaction | β (SE) | 0.746 (0.028) | -1.595 (0.061) | Strong metabolic effect |
| P-value | — | <0.001 | <0.001 | Highly significant |
| Sample size | N subjects/visits | 1,685/9,385 | 1,678/9,250 | — |
| OLS with clustered SE (sensitivity) | Subject-clustered errors | — | — | — |
| Time × FDG interaction | β (SE) | 0.104 (0.042) | -0.059 (0.107) | Attenuated but consistent direction |
| P-value | — | 0.014 | 0.577 | Reduced power with OLS |
| AIC/BIC | — | 49,471/49,500 | 67,996/68,025 | — |
| **Inclusion Criteria Sensitivity:** | | | | |
| ≥3 visits requirement | Excludes short follow-up | — | — | — |
| Time × FDG interaction | β (SE) | 0.104 (0.042) | -0.081 (0.107) | Consistent effect direction |
| p-value | — | 0.016 | 0.461 | Significant for MMSE |
| Sample size | N subjects/visits | 1,426/8,984 | 1,406/8,821 | Reduced sample |
| ≥90 days follow-up | Minimum meaningful follow-up | — | — | — |
| Time × FDG interaction | β (SE) | 0.100 (0.042) | -0.058 (0.107) | Sensitive to follow-up duration |
| P-value | — | 0.018 | 0.593 | Consistent MMSE effect |
| Sample size | N subjects/visits | 1,567/9,266 | 1,563/9,135 | — |
| ≥180 days follow-up (primary) | Preferred analysis sample | — | — | — |
| Time × FDG interaction | β (SE) | 0.100 (0.042) | -0.062 (0.107) | Identical to 90-day filter |
| P-value | — | 0.018 | 0.564 | Stable results |
| Sample size | N subjects/visits | 1,567/9,266 | 1,541/9,091 | — |
| **Nonlinearity Assessment:** | | | | |
| Linear time trend (primary) | Standard linear mixed-effects | — | — | — |
| Model fit | AIC/BIC | 49,471/49,500 | 67,996/68,025 | — |
| Spline time trend (sensitivity) | Flexible nonlinear relationship | — | — | — |
| Model fit | AIC/BIC | 49,267/49,338 | 67,899/67,971 | Better fit with splines |
| Model improvement | ΔAIC/ΔBIC | -205/-162 | -97/-54 | Supports nonlinear relationships |
| **Missing Data Impact:** | | | | |
| **Missing data patterns:** | | | | |
| MMSE missing | % of total visits | 18.3% | — | Moderate missingness |
| ADAS missing | % of total visits | — | 29.3% | Higher missingness |
| FDG missing | % of total visits | 36.2% | 36.2% | High missingness |
| **Included vs excluded comparison:** | | | | |
| Included participants | MMSE mean ± SD | 27.26 ± 2.67 | — | Slightly lower mean |
| Excluded participants | MMSE mean ± SD | 27.31 ± 3.29 | — | Higher variability |
| Included participants | ADAS mean ± SD | — | 16.60 ± 9.58 | Higher mean (more impaired) |
| Excluded participants | ADAS mean ± SD | — | 13.97 ± 9.05 | Lower variability |
| **Cross-Validation:** | | | | |
| 5-fold grouped cross-validation | Subject-level grouping | — | — | — |
| RMSE | Prediction accuracy | 1.43 | 3.00 | Excellent predictive accuracy |
| MAE | Average absolute error | 1.00 | 2.19 | Low prediction errors |
| 95% PI coverage | Calibration quality | 95.0% | 94.7% | Excellent calibration |
| **Effect Size Consistency:** | | | | |
| Primary effect magnitude | MixedLM estimate | 0.746 | -1.595 | Large effect sizes |
| Alternative method difference | — | MixedLM - OLS | — | 0.642 |
| Direction consistency | Across all methods | Positive (protective) | Negative (protective) | Consistent direction |
| Statistical significance | Significance | Mixed-effects: p<0.001 | Mixed-effects: p<0.001 | Highly significant |

***Abbreviations:*** *MMSE, Mini-Mental State Examination; ADAS, Alzheimer's Disease Assessment Scale; FDG, fluorodeoxyglucose positron emission tomography; SE, standard error; OLS, ordinary least squares; AIC, Akaike Information Criterion; BIC, Bayesian Information Criterion; RMSE, root mean square error; MAE, mean absolute error; PI, prediction interval; N, Number.*
