# Supplementary material for: Baseline FDG-PET Brain hypometabolism as a predictive biomarker of cognitive decline and Alzheimer’s disease risk
Source: J Nutr Health Aging. 2026 Mar 11;30(5):100823. doi: 10.1016/j.jnha.2026.100823 (PMC12994019; doi:10.1016/j.jnha.2026.100823)
Supplement: Supplementary file 7 [file mmc7.docx]

**Supplementary Table 7:** Metabolic Biomarker Implementation, Thresholds and Comparative Performance.

| **Implementation Component** | **Specification** | **Performance Metric** | **Application** |
| --- | --- | --- | --- |
| **FDG PET Biomarker Thresholds:** | | | |
| Low risk (preserved metabolism) | FDG z-score >0.5 | 24m AD risk: 6.3% | Standard monitoring (every 2-3 years) |
| Low risk (preserved metabolism) | FDG z-score >0.5 | 24m MCI risk: 44.6% | Lifestyle counseling |
| Intermediate risk (mild hypometabolism) | FDG z-score -0.5 to 0.5 | 24m AD risk: 12.6% | Enhanced monitoring (every 12-18 months) |
| Intermediate risk (mild hypometabolism) | FDG z-score -0.5 to 0.5 | 24m MCI risk: 45.8% | Intensive lifestyle + consider trials |
| High risk (severe hypometabolism) | FDG z-score <-0.5 | 24m AD risk: 23.6% | Intensive monitoring (every 6-12 months) |
| High risk (severe hypometabolism) | FDG z-score <-0.5 | 24m MCI risk: 46.9% | Clinical trials + multi-modal intervention |
| **Predictive Accuracy (AUC) by Timeframe:** | | | |
| **AD conversion prediction:** | | | |
| 12 months | FDG-based model | 0.812 | Good discrimination |
| 24 months | FDG-based model | 0.826 | Good discrimination |
| 36 months | FDG-based model | 0.819 | Good discrimination |
| **MCI conversion prediction:** | | | |
| 12 months | FDG-based model | 0.686 | Acceptable discrimination |
| 24 months | FDG-based model | 0.643 | Acceptable discrimination |
| 36 months | FDG-based model | 0.680 | Acceptable discrimination |
| **Model Calibration (Brier Score):** | | | |
| AD conversion | 0.089-0.092 | Good calibration | Reliable probability estimates |
| MCI conversion | 0.239-0.244 | Acceptable calibration | Reliable probability estimates |
| **Comparative Biomarker Performance:** | | | |
| **FDG metabolic effect size:** | | | |
| MMSE decline | β = 1.364 (SE 0.059) | p<0.001 | Large effect size |
| ADAS worsening | β = -5.311 (SE 0.198) | p<0.001 | Large effect size |
| **Time × FDG interaction strength:** | | | |
| MMSE decline | β = 0.746 (SE 0.028) | p<0.001 | Strong modulation effect |
| ADAS worsening | β = -1.595 (SE 0.061) | p<0.001 | Strong modulation effect |
| **Model Significance Across Inclusion Criteria:** | | | |
| **MMSE assessment:** | | | |
| ≥3 visits requirement | β = 0.104, p = 0.016 | Consistent effect | Robust to selection criteria |
| ≥90 days follow-up | β = 0.100, p = 0.018 | Consistent effect | Robust to follow-up duration |
| ≥180 days follow-up | β = 0.100, p = 0.018 | Consistent effect | Robust to follow-up duration |
| **Implementation Framework:** | | | |
| Target population | CN individuals ≥65 years | — | Primary prevention focus |
| Screening method | FDG PET with MetaROI analysis | — | Standardized acquisition protocol |
| Risk stratification approach | Tertile-based FDG z-scores | — | Simple clinical decision rule |
| **Therapeutic Stratification:** | | | |
| **Trial enrichment strategy:** | | | |
| Prevention trials | FDG z-score >0 | Lower baseline risk | Longer trial duration needed |
| Early intervention trials | FDG z-score -1 to 0 | Moderate baseline risk | Optimal risk-benefit ratio |
| Symptomatic trials | FDG z-score <-1 | High baseline risk | Shorter time to endpoints |
| **Decision Thresholds:** | | | |
| High-risk MCI conversion flag | Predicted probability ≥30% | Sensitivity ~65% | Triggers enhanced monitoring |
| High-risk AD conversion flag | Predicted probability ≥10% | Sensitivity ~71% | Triggers intensive intervention |
| **Biomarker Advantages Over Standard Care:** | | | |
| Early detection capability | Preclinical identification | Years before symptoms | Expanded therapeutic window |
| Prognostic precision | Quantitative risk estimates | Individualized counseling | Patient-specific management |
| Treatment monitoring potential | Serial FDG assessments | Objective treatment response | Biomarker-guided therapy |
| Trial efficiency enhancement | Enriched populations | Reduced sample sizes | Accelerated drug development |

***Abbreviations:*** *FDG, fluorodeoxyglucose positron emission tomography; CN, cognitively normal; MCI, mild cognitive impairment; AD, Alzheimer's disease; MMSE, Mini-Mental State Examination; ADAS, Alzheimer's Disease Assessment Scale; MetaROI, meta-region of interest composite score; AUC, area under curve; SE, standard error.*
